# Utility of CT-based navigation in revision total hip arthroplasty for a patient with severe posterior pelvic tilt-case report-

**DOI:** 10.1186/s12891-020-03263-9

**Published:** 2020-04-16

**Authors:** Shintaro Watanabe, Hyonmin Choe, Naomi Kobayashi, Hiroyuki Ike, Daigo Kobayashi, Syota Higashihira, Yutaka Inaba

**Affiliations:** Department of Orthopedic Surgery, Yokohama City University, 3-9 Fukuura, Kanazawa-ku, Yokohama City, Kanagawa Japan

**Keywords:** Posterior pelvic tilt, Navigation, Total hip arthroplasty

## Abstract

**Background:**

Hip dislocation after total hip arthroplasty (THA) or hemi-arthroplasty is a rare but serious complication. Dislocation may be prevented by appropriate positioning of the cup angle of inclination and anteversion.

**Case presentation:**

This report describes a 66-year-old woman who underwent revision THA using a computer tomography (CT)-based navigation system to treat an anterior dislocation after hemi-arthroplasty due to a severe posterior pelvic tilt. At initial presentation, her sagittal pelvic tilt angle, measured as anterior pelvic plane (APP) in the supine position, was 38 degrees posterior to the coronal plane. Owing to the posterior pelvic tilt, revision THA was performed using CT-based navigation, while dual mobility was utilized to reduce the risk of re-dislocation. Postoperatively, her sagittal pelvic tilt angle showed further progression over time, with an APP of 66 degrees posterior to the coronal plane in the standing position 3 years after revision THA. Simulation with the Zed Hip system showed that the risk of implant-to-implant impingement was much higher posteriorly than anteriorly. Gait analysis demonstrated hyperextension of the hip joint while walking, although hip joint function required for daily activity was maintained.

**Conclusions:**

Preoperative planning of implant orientation, based on posterior progression of pelvic tilt and accurate placement of components, is important to prevent dislocation in patients with severe posterior pelvic tilt. A dual mobility cup may also improve hip function in these patients.

## Background

Changes in sagittal pelvic tilt affect the functional angle of implant position after total hip arthroplasty (THA). Appropriate positioning of the acetabular component can reduce the risk of hip dislocation after THA. Consideration of changes in functional cup inclination and anteversion due to sagittal pelvic tilt is critical in obtaining good clinical outcomes [1].

This report describes a patient who underwent revision THA using a computer tomography (CT)-based navigation system to treat an anterior dislocation after hemi-arthroplasty caused by a severe posterior pelvic tilt. Use of a CT-based navigation system enabled accurate cup placement in the presence of a severe posterior pelvic tilt. Daily activities of the patient were preserved despite a progressive posterior change in pelvic tilt after revision THA.

## Case presentation

A 66-year-old woman presented at our hospital for the treatment of a dislocation 15 years after hemi-arthroplasty. She had undergone hemi-arthroplasty at another hospital using a porous coated anatomical stem (Stryker, MI) for a femoral neck fracture. The patient had poor posture due to compression fracture but was still able to walk until 4 months before presentation at our hospital. The patient had difficulty walking without experiencing trauma and was diagnosed with an anterior dislocation due to reduction of lumbar lordosis and severe posterior pelvic tilt. Because the dislocation could not be reduced at the other hospital, the patient was admitted to our hospital for treatment.

**Fig. 1:**
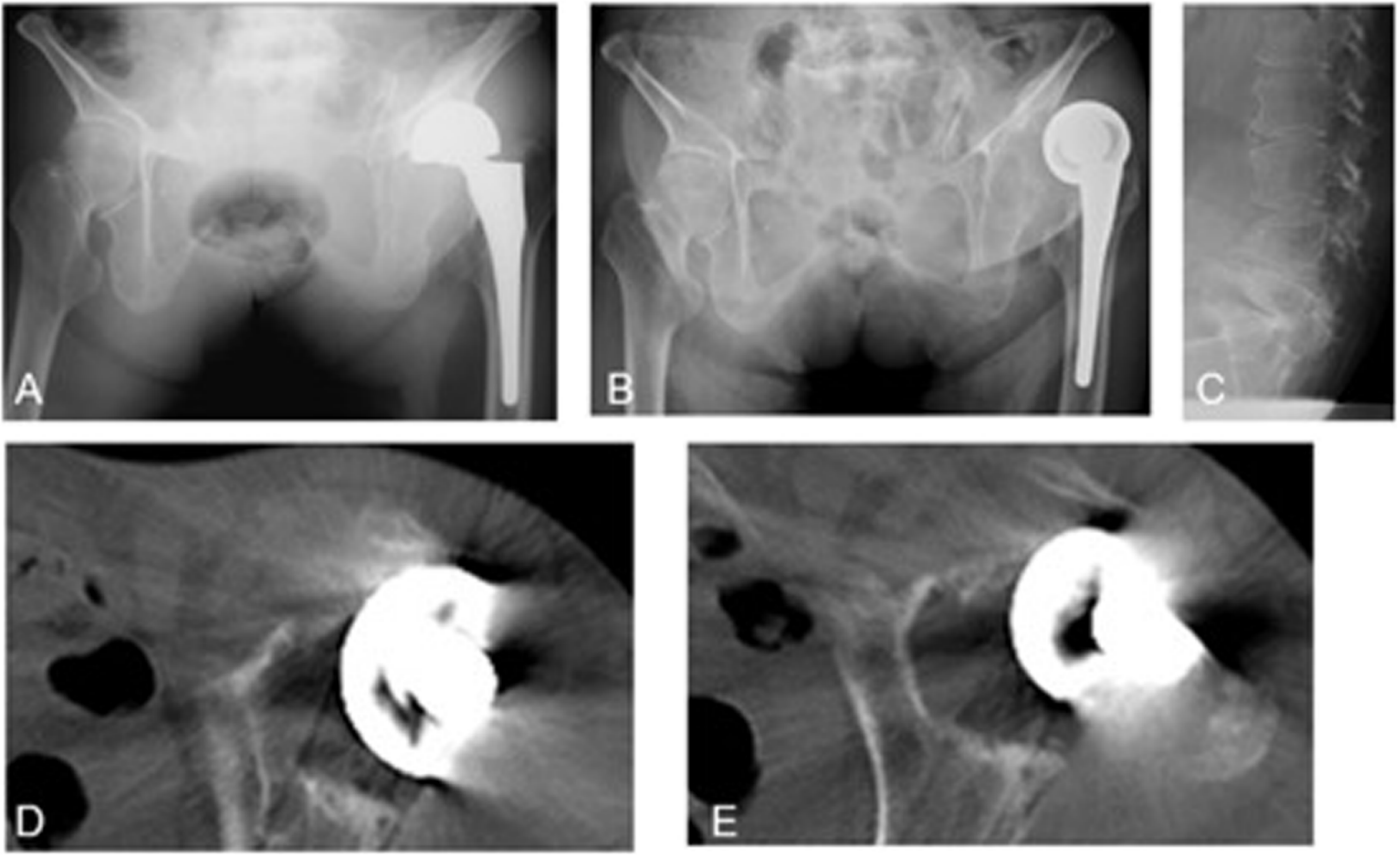
Preoperative hip X-rays and CT images. **a** X-ray image of the front of the hip with the patient in a standing position, taken at a previous hospital 1 year earlier, showing severe posterior pelvic tilt. **b** X-ray image of the front of the hip with the patient in a supine position at first visit to our hospital, showing anterior dislocation of the hemi-arthroplasty and a posterior pelvic tilt. **c** Lumbar sagittal X-ray image of the patient in the supine position at first visit to our hospital, showing a wedge-shaped compression fracture of the fifth lumbar vertebra and lumbar lordosis. **d** Preoperative axial CT images, showing a small bone fragment, which suggested an acetabulum anterior bone fracture

Physical findings at the time of her first visit to our hospital included height of 148 cm, weight of 38 kg, and body mass index of 17.4 kg/m^2^. There was no tenderness at the femoral triangle, her left leg was shortened in external rotation, she had limited range of motion (ROM; 70 ° of flexion, − 10 ° of extension and 10 ° of abduction) in her left hip joint, and she was unable to keep standing. An X-ray image of her hip in the supine position showed anterior dislocation of the hemi-arthroplasty with severe posterior pelvic tilt (Fig. 1). An X-ray image of her lumbar spine showed a wedge-shaped compression fracture of the fifth lumbar vertebra due to osteoporosis and reduction of lumbar lordosis. The small bone fragment in the anterior part of the acetabulum indicated the occurrence of a dislocation fracture due to stress concentration on the anterior wall of the acetabulum.

The anterior pelvic plane (APP), defined as a plane through the bilateral anterior superior iliac spines to the pubic symphysis, was regarded as the anatomic plane of the pelvis in this patient. The angle between the APP and the coronal plane passing through the bilateral anterior superior iliac spines (ASIS) was defined as the sagittal pelvic tilt angle (APP tilting angle) in the supine or standing position. CT data and the Zed Hip system (Lexi, Japan) showed that the APP tilting angle in this patient at admission was 38 degrees posterior in the supine position.

**Fig. 2:**
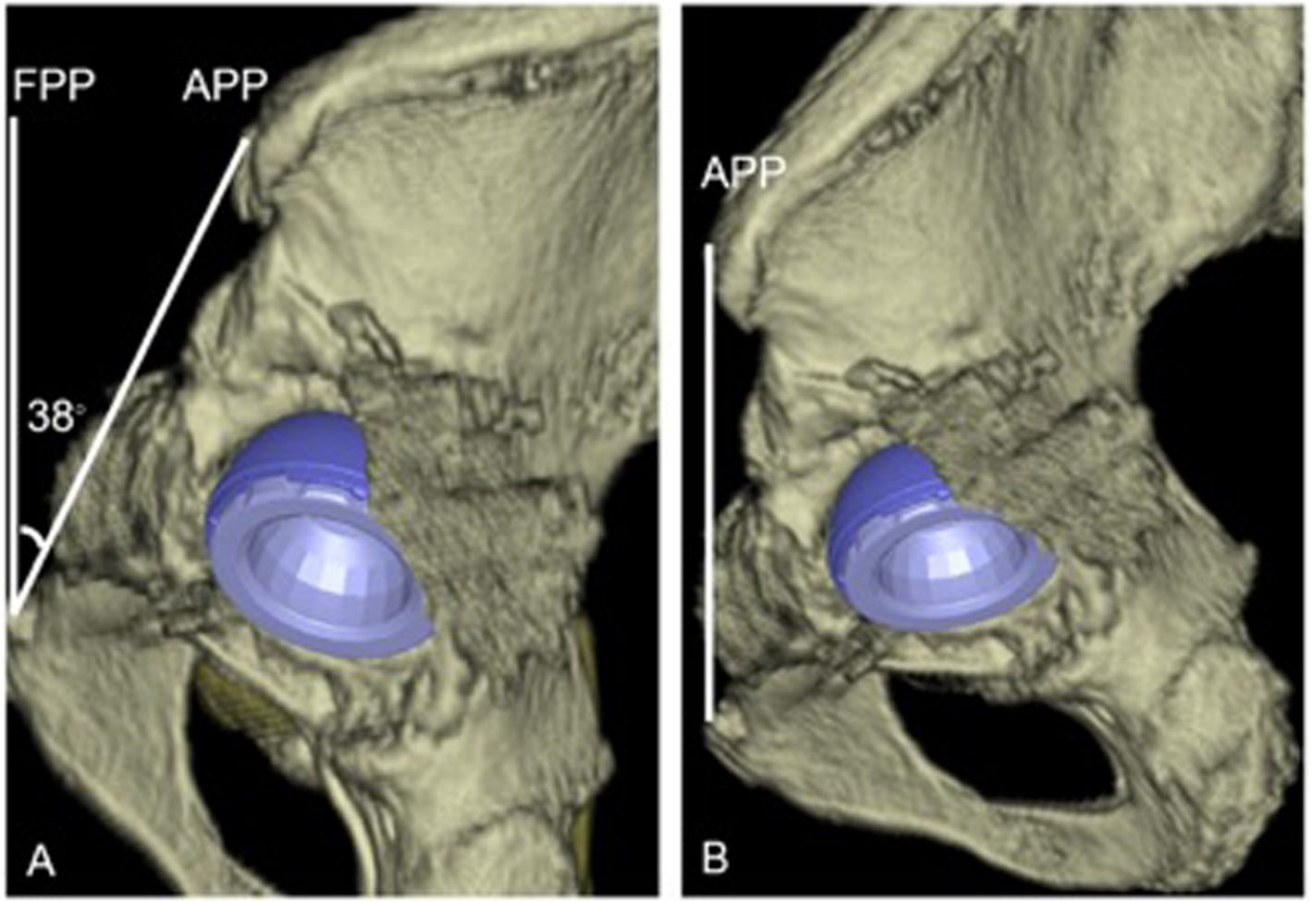
Preoperative planning of cup placement on 3D images. **a** Lateral view of the pelvis with the cup in the horizontal plane. The APP tilting angle in the supine position on CT images was 38 degrees retroversion for the horizontal plane. Because of stem anteversion, the inclination was planned radiographically to be 40 degrees and the anteversion was planned to be 26 degrees relative to the FPP. **b** Lateral view of the pelvis with the cup in the APP, showing a cup angle of inclination of 37 degrees and a cup angle of anteversion of − 5 degrees relative to the APP

We planned to transform hemi-arthroplasty into THA because anterior coverage with reduced cup anteversion was required for the prevention of anterior dislocation in this case. Due to the severe posterior tilting of the pelvis, cup positioning was planned preoperatively (Fig. 2). Because there was no evidence suggesting the loosening of the femoral component, the stem was planned to be retained. CT showed that the anteversion angle of the stem on the femoral table top plane was 16 degrees. Based on a previously published method of calculation [2, 3], we planned radiographic cup positioning angle to be 40 degrees of inclination and 26 degrees of anteversion relative to the functional pelvic plane (FPP), which was defined as the coronal plane passing through the bilateral ASIS in the supine position. The anatomic cup positioning angle was planned to be 37.3 degrees of inclination and − 8.3 degrees of anteversion, whereas the radiographic cup positioning angle was planned to be 37 degrees of inclination and − 5 degrees of anteversion and the operative cup positioning angle was planned to be 36.8 degrees of inclination and − 6.3 degrees of anteversion, relative to the APP based on a previously published method [4]. Preoperative planning using the Stryker navigation system (Stryker, MI) [2, 4], a CT-based hip navigation system (Fig. 2). We did not perform ROM simulation before surgery because the patient’s implant was already dislocated at the presence to our hospital and the CT data cannot to be used for the ROM simulation.

The operation was performed using a direct lateral approach. The stem was retained after confirmation of rigid stability during surgery. Exposure of the acetabulum revealed a bone defect in its anterior wall. A 46 mm cup was inserted at 37 degrees of inclination and − 5 degrees of anteversion relative to the APP, as determined radiographically using navigation. Because some patients experience progressive posterior changes in pelvic tilt after surgery, the dual mobility MDM X3 Mobile Bearing Hip System (Stryker, MI) was utilized to reduce the risk of re-dislocation. Intraoperative press fit on acetabular implant was adequate for initial stability.

**Fig. 3:**
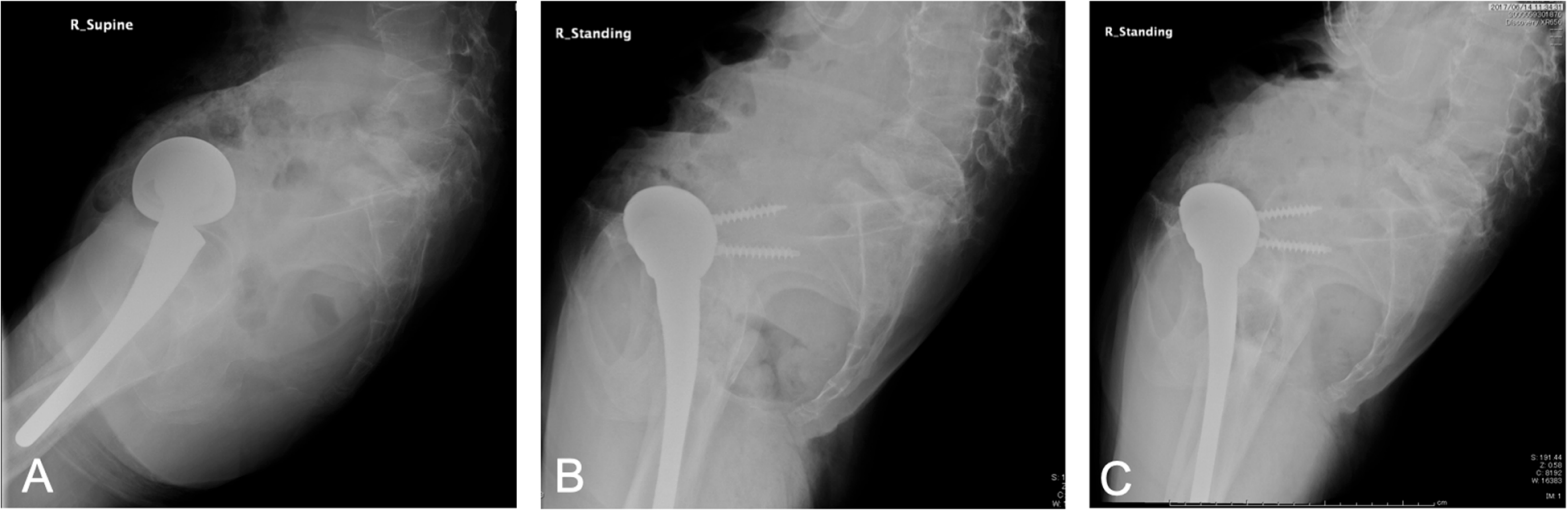
X-ray lateral images of the pelvis, showing the time course of posterior changes in the pelvic tilting angle. Lateral pelvic X-ray images in the supine position preoperatively (**a**) and in the standing position 1 year (**b**) and 2 years (**c**) postoperatively

**Fig. 4:**
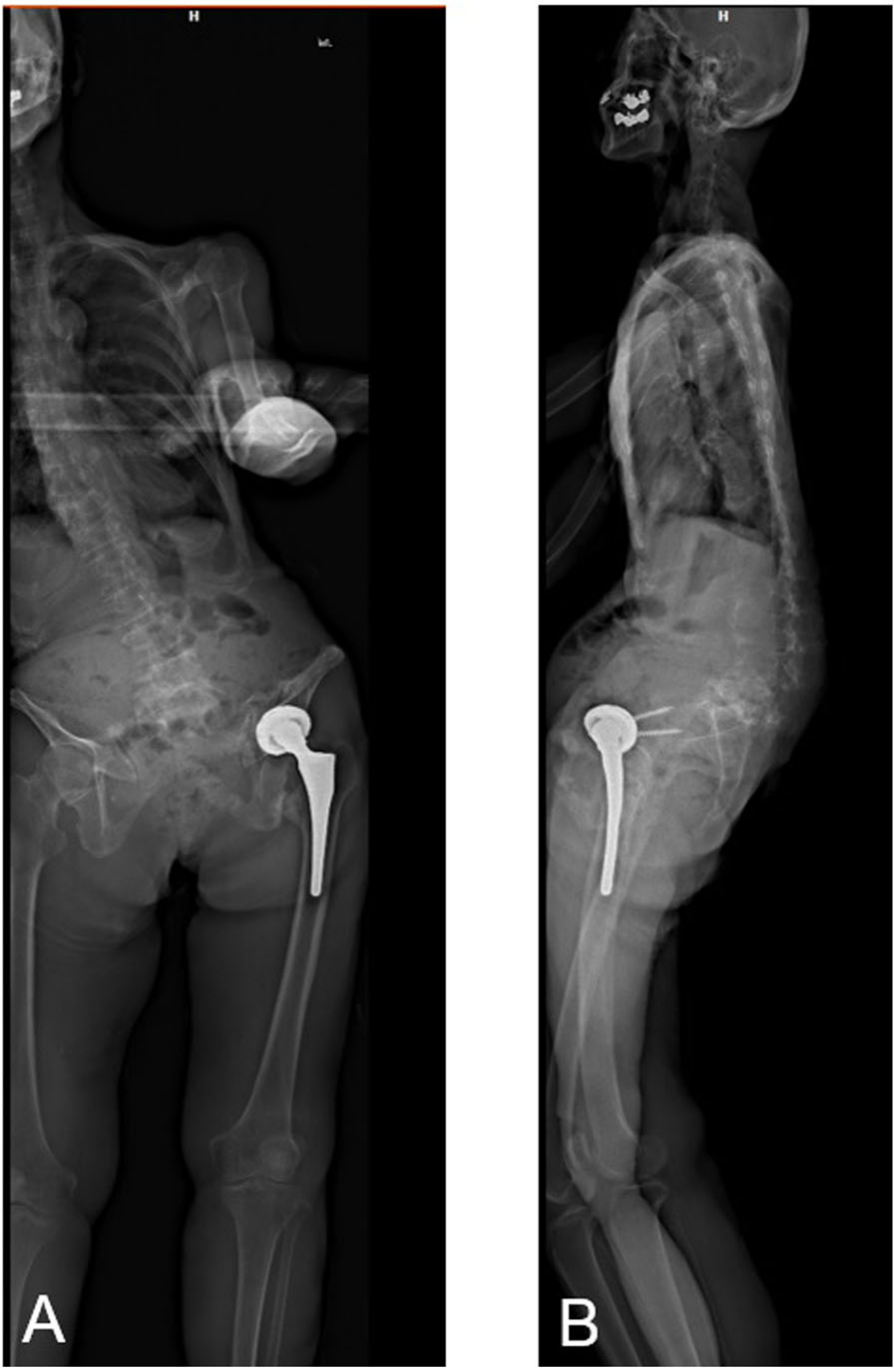
EOS imaging system of the pelvis 3 years after surgery. Coronal (**a**) and sagittal (**b**) images. The APP tilting angle was measured as 60 degrees posterior to the vertical plane in the standing position

**Fig. 5:**
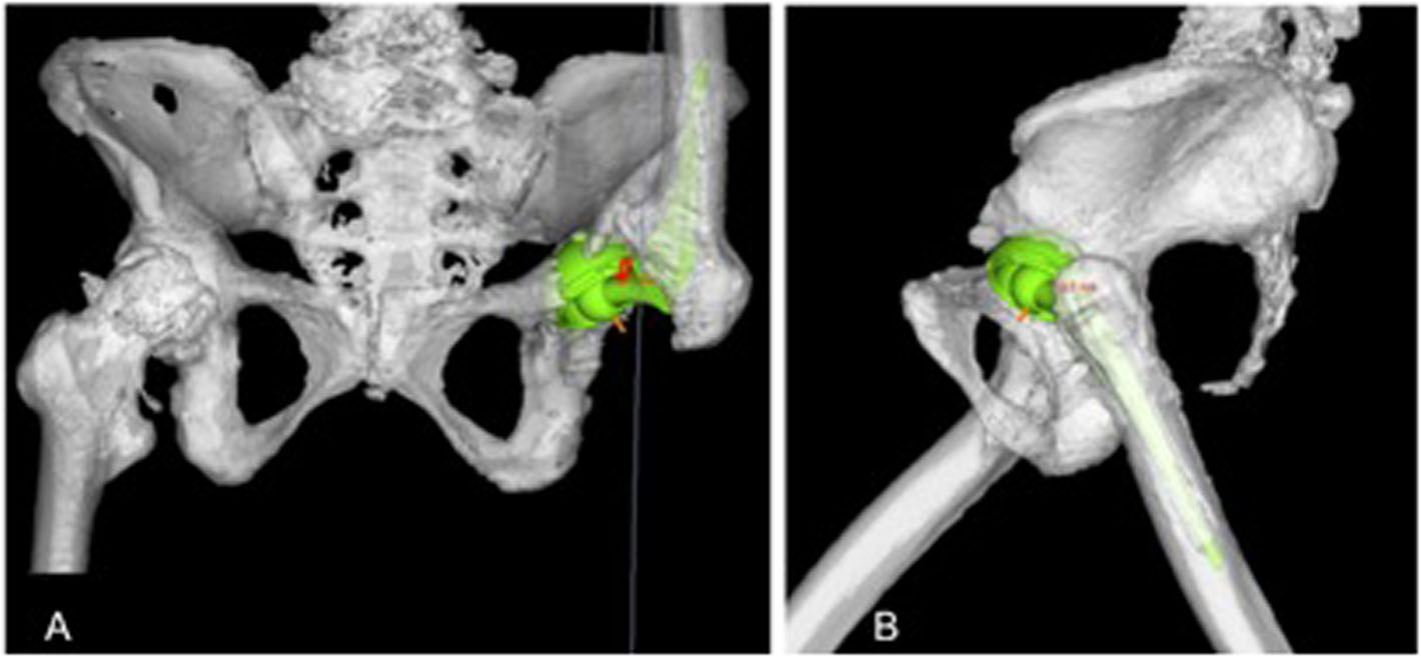
ROM simulation using Zed Hip after THA. **a** Coronal and **b** sagittal images of ROM simulation using Zed Hip after THA, showing implant-to-implant impingement at a flexion of 99 degrees and an extension of 68 degrees relative to the APP in this patient

**Fig. 6:**
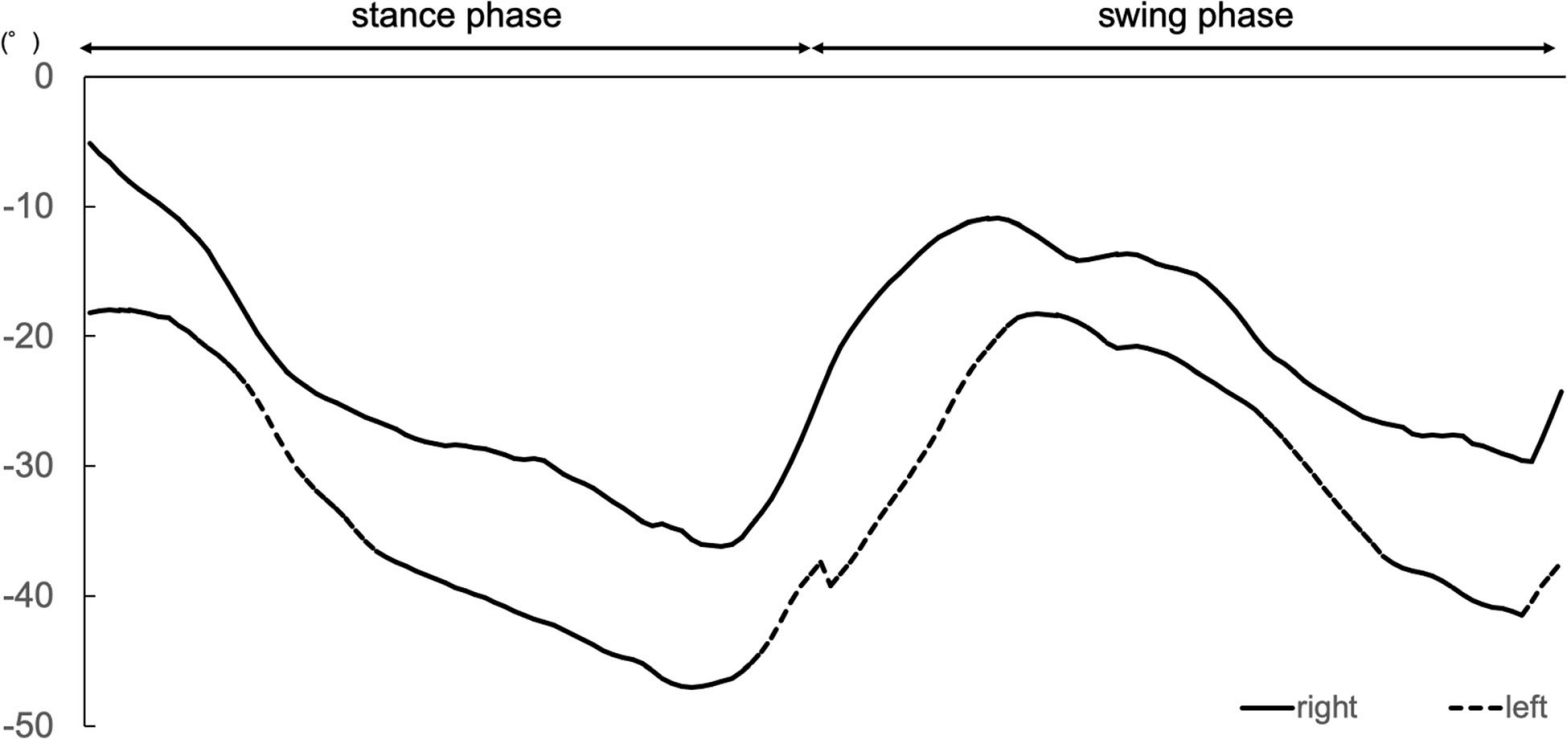
Gait analysis of hip joint angle 3 years after surgery. The angle of the left hip joint ranged from a maximum of − 18 degrees to a minimum of − 47 degrees. Gait analysis showed hyperextension of the left hip joint relative to the right hip

The patient started standing and walking rehabilitation the day after the operation. Three months later, she was able to walk short distances without pain and had a Harris Hip score (HHS) of 78 points. Measurement of her APP tilting angle showed progressive posterior pelvic tilting after the operation (Fig. 3), with CT 1 year later showing an APP tilting angle of 47 degrees in the supine position. Three years after the operation, EOS imaging (Paris, France) showed that her APP tilting angle was 60 degrees posterior in the standing position (Fig. 4). The sagittal view of EOS imaging showed the angle between APP and femoral shaft was measured to be − 66 degrees (66 degrees extension), indicating hyperextension of the femur in the standing position. The maximum ROM of this patient was assessed by the Zed Hip system. Implant-to-implant impingement was observed at a flexion of 99 degrees and an extension of 68 degrees relative to the APP (Fig. 5), indicating a high risk of posterior impingement in the standing position. Gait analysis was also performed 3 years after surgery using VICON612 (Vicon Motion Systems, Oxford, UK) and plug-in-gait marker placement [5]. The hip joint angle was defined as the angle between the APP and the femoral bone axis. The left hip joint was hyperextended relative to the right hip (Fig. 6), although the patient was still able to walk for short distances without pain. At the final follow up of three years after operation, hip flexion, extension, inner rotation and external rotation angle and HHS were improved to 90 °, 0 °, 20 ° and 0 °, and was 74 points.

## Discussion

Hip dislocation after THA or hemi-arthroplasty is a rare but serious complication. Dislocation may be prevented by appropriate positioning of the cup angle of inclination and anteversion [2, 6–8]. Because sagittal pelvic tilt significantly affects functional cup inclination and anteversion, it is necessary to consider changes of pelvic tilt for accurate cup placement. Although most patients who undergo THA do not experience postural changes in pelvic tilt, some patients may experience severe changes in pelvic tilt from the supine to the standing position, which may result in anterior dislocation following THA [9]. In most patients, the coronal plane in the supine position can act as the FPP for planning of the cup angle [9]. THA patients with small lumbo-lordotic angle have tendency of time course change of posterior pelvic tilt after surgery [1, 3]. In this case, because the patient had a reduced-lumbar lordosis, postoperative posterior pelvic change was expected and then, the change was considered in preoperative planning of cup positioning [1, 3].

Our report describes a patient who experienced anterior dislocation after hemi-arthroplasty. Previously, less than − 10 ° of APP angle was considered to increase the anterior dislocation in standing position [3], therefore, our current patient was considered having the severe posterior pelvic tilt that resulted in anterior dislocation. In the previous reports, late postoperative dislocation of THA due to severe posterior tilt were treated by revision THA with elevated liner or optimization of cup anteversion [10] or use of dual mobility cup [11]. In our case, she underwent revision THA using a CT-based navigation system for the appropriate cup positioning in combination with a dual mobility cup. In this patient, the coronal plane in the supine position was considered the FPP, because she had difficulty standing due to hip dislocation and had a severe posterior pelvic tilt in the supine position. Thus, radiographic preoperative planning of cup placement resulted in an inclination of 40 degrees and an anteversion of 26 degrees relative to the FPP (the coronal plane in the supine position), whereas anatomic preoperative planning of cup placement resulted in an inclination of 37.3 degrees and an anteversion of − 8.3 degrees relative to the APP. A dual mobility cup was utilized in this patient to prevent dislocation due to progressive posterior changes in pelvic tilt. This patient experienced a progressive posterior change in pelvic tilt after the operation from the supine position preoperatively to the standing position postoperatively. Interestingly, the change in pelvic tilt resulted in hyperextension of the femur, as she did not experience a compensational change in knee flexion.

Evaluation of global alignment using an EOS imaging system showed that the extension angle of the femur was 66 degrees relative to the APP, indicating an increased risk of bone-to-bone or implant-to-implant impingement in the standing position. This may have been due to the extension of ROM resulting from reduced cup anteversion, the surgical removal of the impinging bone, and/or the surgical improvement in contractions. ROM simulation using the Zed Hip system indicated that femoral extension of 68 degrees relative to the APP resulted in implant-to-implant impingement. Gait analysis demonstrated femoral hyperextension while walking. These results indicated that the risk of posterior impingement was high in this patient.

CT-based navigation was reportedly useful tool for the accurate placement of implants during THA [12, 13]. Appropriate implant positioning is important in preventing dislocation, therefore, cup planning should be planned considering the progressive posterior change of pelvic tilt in patients with severe posterior pelvic tilt.

We utilized dual mobility cup in the current case, because dual mobility cups reportedly reduce dislocation rate after THA [11, 14]. Although dual mobility cup may not be suitable for young patients or re-revision cases [15], it may be a useful option for the prevention of dislocation for patients with severe posterior pelvic tilt.

## Conclusion

This report describes a patient who underwent THA using CT-based navigation for dislocation after hemi-arthroplasty with a posterior pelvic tilt. Implant planning in patients with preoperative posterior pelvic tilt should consider possible progressive postoperative posterior changes in pelvic tilt while standing. Dual mobility cups may be useful in patients at risk of dislocation.

## Data Availability

All the data supporting our findings are contained within the manuscript.

## Abbreviations

THA: Total hip arthroplasty
CT: Computer tomography
ROM: Range of motion
APP: Anterior pelvic plane
FPP: Functional pelvic plane
HHS: Harris Hip score.

## References

[1] Suzuki H, Inaba Y, Kobayashi N, Ishida T, Ike H, Saito T (2016). Postural and chronological change in pelvic tilt five years after Total hip Arthroplasty in patients with developmental dysplasia of the hip: a three-dimensional analysis. J Arthroplast.

[2] Widmer KH, Zurfluh B (2004). Compliant positioning of total hip components for optimal range of motion. J Orthop Res.

[3] Inaba Y, Kobayashi N, Suzuki H, Ike H, Kubota S, Saito T (2016). Preoperative planning for implant placement with consideration of pelvic tilt in total hip arthroplasty: postoperative efficacy evaluation. BMC Musculoskelet Disord.

[4] Murray DW (1993). The definition and measurement of acetabular orientation. J Bone Joint Surg Br Vol.

[5] Davis RB, OS, Tyburski D, Gage JR (1991). A gait analysis data collection and reduction technique. Hum Mov Sci.

[6] Yoshimine F (2006). The safe-zones for combined cup and neck anteversions that fulfill the essential range of motion and their optimum combination in total hip replacements. J Biomech.

[7] Lewinnek GE, Lewis JL, Tarr R, Compere CL, Zimmerman JR (1978). Dislocations after total hip-replacement arthroplasties. J Bone Joint Surg Am.

[8] Dorr LD, Malik A, Dastane M, Wan Z (2009). Combined anteversion technique for total hip arthroplasty. Clin Orthop Relat Res.

[9] Nishihara S, Sugano N, Nishii T, Ohzono K, Yoshikawa H. Measurements of pelvic flexion angle using three-dimensional computed tomography. Clin Orthop Relat Res. 2003;411:140–51. 10.1097/01.blo.0000069891.31220.fd 12782869

[10] Kobayashi H, Nakashima Y, Yamamoto T (2016). Late anterior dislocation due to posterior pelvic tilt in Total hip Arthroplasty. Open Orthop J.

[11] Carulli C, Macera A, Matassi F, Civinini R, Innocenti M (2016). The use of a dual mobility cup in the management of recurrent dislocations of hip hemiarthroplasty. J Orthop Traumatol.

[12] Kalteis T, Handel M, Bathis H, Perlick L, Tingart M, Grifka J (2006). Imageless navigation for insertion of the acetabular component in total hip arthroplasty: is it as accurate as CT-based navigation?. J Bone Joint Surg Br Vol.

[13] Gurgel HM, Croci AT, Cabrita HA, Vicente JR, Leonhardt MC, Rodrigues JC (2014). Acetabular component positioning in total hip arthroplasty with and without a computer-assisted system: a prospective, randomized and controlled study. J Arthroplast.

[14] Epinette JA (2015). Clinical outcomes, survivorship and adverse events with mobile-bearings versus fixed-bearings in hip arthroplasty-a prospective comparative cohort study of 143 ADM versus 130 trident cups at 2 to 6-year follow-up. J Arthroplast.

[15] Hailer NP, Weiss RJ, Stark A, Karrholm J (2012). Dual-mobility cups for revision due to instability are associated with a low rate of re-revisions due to dislocation: 228 patients from the Swedish hip Arthroplasty register. Acta Orthop.

